# The Relations of Attention to and Clarity of Feelings With Facial Affect Perception

**DOI:** 10.3389/fpsyg.2022.819902

**Published:** 2022-07-06

**Authors:** Thomas Suslow, Anette Kersting

**Affiliations:** Department of Psychosomatic Medicine and Psychotherapy, University of Leipzig Medical Center, Leipzig, Germany

**Keywords:** attention to emotions, clarity of emotions, perception of facial emotions, schematic faces, negative interpretative bias

## Abstract

Attention to emotions and emotional clarity are core dimensions of individual differences in emotion awareness. Findings from prior research based on self-report indicate that attention to and recognition of one’s own emotions are related to attention to and recognition of other people’s emotions. In the present experimental study, we examined the relations of attention to and clarity of emotions with the efficiency of facial affect perception. Moreover, it was explored whether attention to and clarity of emotions are linked to negative interpretations of facial expressions. A perception of facial expressions (PFE) task based on schematic faces with neutral, ambiguous, or unambiguous emotional expressions and a gender decision task were administered to healthy individuals along with measures of emotion awareness, state and trait anxiety, depression, and verbal intelligence. Participants had to decide how much the faces express six basic affects. Evaluative ratings and decision latencies were analyzed. Attention to feelings was negatively correlated with evaluative decision latency, whereas clarity of feelings was not related to decision latency in the PFE task. Attention to feelings was positively correlated with the perception of negative affects in ambiguous faces. Attention to feelings and emotional clarity were not related to gender decision latency. According to our results, dispositional attention to feelings goes along with an enhanced efficiency of facial affect perception. Habitually paying attention to one’s own emotions may facilitate processing of external emotional information. Preliminary evidence was obtained suggesting a relationship of dispositional attention to feelings with negative interpretations of facial expressions.

## Introduction

Two central dimensions of stabile individual differences in emotional awareness are attention to emotions and emotional clarity (Palmieri et al., 2009). Attention to emotions refers to focusing one’s attention on emotional processes, and becoming aware of and valuing them, whereas emotional clarity relates to meta-knowledge about emotions, the ability to unambiguously recognize, label, and characterize one’s emotions (Boden and Thompson, 2017). Past research on attention to and clarity of emotions has primarily examined their trait or dispositional forms, but both are dynamic constructs that temporally vary and that can also be assessed at the state level (Thompson and Boden, 2019). Attention to and clarity of emotions can be distinguished from two other dimensions of emotional awareness, the magnitude with which individuals tend to experience emotions (the intensity dimension), and the tendency to outwardly express emotions (the expression dimension; Gohm and Clore, 2002). Attention to emotions and emotional clarity are not independent of each other but appear to be moderately positively associated (Boden and Thompson, 2017). An increased habitual attention to one’s emotions could help at least to some degree to gain or improve emotional clarity over time. There is evidence of gender differences concerning attention to emotions but not emotional clarity. Women appear to attend more to their emotions than men whereas they seem to be equal to men in the extent to which they identify, label, and represent the type of emotion experienced (Boden and Berenbaum, 2012; Boden et al., 2013a; Mankus et al., 2016).

Emotion perception can be defined as processes that comprise the identification of emotional information in the environment or the self (Phillips, 2003; Beer and Ochsner, 2006). In the past, research on exteroceptive emotion processing had a focus on the visual modality and in particular on the perception of facial expressions (PFE; Schirmer and Adolphs, 2017). The correct recognition of emotions in other people’s faces is essential for successful social interaction (Ferretti and Papaleo, 2019). Results from tasks requiring an explicit categorization of emotional facial expressions indicate that, among the basic emotions, the expression of happiness is recognized faster and more accurately than the other emotions, whereas facial fear is recognized more slowly and less accurately than the other expressions (Palermo and Coltheart, 2004; Kosonogov and Titova, 2019). Findings from meta-analyses on gender differences in facial emotion recognition indicate a small to moderate female advantage (Hall, 1978; McClure, 2000). Even though there is evidence that the emotional quality of facial expressions can be identified rapidly and even outside of conscious awareness (e.g., Rohr et al., 2012), it is assumed that these early perceptual processes influence only to a minor extent the explicit categorical recognition of facial expressions which includes semantic or conceptual knowledge and contextual information (Calvo and Nummenmaa, 2016).

Attention can be conceptualized as a mechanism for selection of information to be perceived with priority (Chun et al., 2011). Selective attention is assumed to enhance perceptual processing of external sensory or internally generated stimuli. Attention allocation to diagnostically relevant facial features such as the eyes and the mouth could improve the decoding of emotional facial expressions (Palermo and Rhodes, 2007; Schurgin et al., 2014). In case of multiple stimuli, attentional resources are directed preferentially and efficiently toward emotional information such as negative or positive pictures (Nummenmaa et al., 2006). Differences in attention allocation to emotional stimuli might be a result of differences in stimulus appraisal or threat evaluation (Yiend, 2010).

Emotional trait predispositions affect and bias the deployment of attention, leading to greater processing resources being directed toward the perception of emotion congruent material. For example, individuals high in trait anxiety and those at risk of developing anxiety disorders have robust attention biases to threatening information, among them facilitated engagement, difficulty in disengaging and later avoidance of threat (Koster et al., 2005; Okon-Singer, 2018). Highly trait anxious individuals were found to recognize fear but not other emotions from faces better than individuals low in trait anxiety (Surcinelli et al., 2006) and to manifest mood-congruent biases in the interpretation of ambiguous facial expressions (Blanchette et al., 2007). Depressed mood has been found to go along with a sad interpretation bias in the perception of ambiguous facial expressions (Beevers et al., 2009). A recent study based on schematic emotional faces revealed differences between high depressiveness and high trait anxiety in working memory for and processing of facial expressions (Tamm et al., 2020): high depressiveness seems to be associated with a lower accuracy for visual encoding of happy faces compared to high trait anxiety.

According to self-report data, recognition of and attention to one’s own emotions are substantially related to recognition of and attention to other people’s emotions (Lischetzke et al., 2001, 2012). Findings from a recent study show that the ability to express one’s feelings in words with a high degree of differentiation goes along with a more accurate decoding of others’ emotional expressions (Israelashvili et al., 2019). This association between self and other perception may result from the fact that both processes depend on the same conceptual emotion knowledge. It can also be argued that the recognition of other individuals’ emotional expressions draws on somatic imitation and simulation processes which are an important part of the bodily reenactment of the experience of the other’s state (Niedenthal, 2007). The correct decoding and understanding of the emotional reactions elicited by others require the ability to recognize one’s own emotions. Interestingly, similar brain regions seem to be involved in recognizing one’s own emotions and those of others (Heberlein and Adolphs, 2007; Heberlein and Atkinson, 2009).

Thus far only few studies have reported on the relations of dispositional attention to and dispositional clarity of emotions with abilities to perceive emotions in others or external environment stimuli as measured by experimental tasks. In an investigation using the emotional Stroop task attention to emotions (but not clarity of emotions) was related to an enhanced attention allocation to lexical emotional stimuli (Coffey et al., 2003). Lexical emotional stimuli are written or spoken words that denote emotional states, moods, or feelings (e.g., “joy”) or that have emotional connotations although they do not directly refer to emotions themselves (e.g., “murder”; Vinson et al., 2014; Chen et al., 2016). Thus, individuals who devote greater attention to their emotions in everyday life seem to dedicate more attention to emotional words in the external world. In an eye-tracking study in which multiple emotional and neutral images were shown simultaneously attention to feelings (but not clarity of feelings) was found to be negatively correlated with entry times of gaze for emotional pictures (Bujanow et al., 2020). These data suggest that devoting habitually attention to one’s emotions is linked to a fast initial orientation of attention toward emotional scenes, irrespective of affective valence. It is assumed that dispositional attention to feelings could go along with a more efficient detection of emotional information in the visual fields and/or with a faster automatic engagement of the oculomotor system toward emotional content (Bujanow et al., 2020). Finally, results of an investigation with an emotional Stroop task in which event-related brain potentials were analyzed indicate that dispositional attention to emotions is associated with heightened early attention to lexical emotional stimuli regardless of affective valence (Fisher et al., 2010). All in all, there is preliminary evidence that dispositional attention to one’s feelings might be linked to a more intensified early processing of and a more rapid allocation of attentional resources to external emotional information. It remains to be further examined whether dispositional emotional attention could facilitate processing of external emotional information and accelerate evaluative decisions about external emotional stimuli.

Although theoretically it seems plausible that dispositional clarity concerning one’s own emotions should be positively associated with the identification and labeling of other people’s emotions and self-report data confirm this idea (Lischetzke et al., 2001, 2012) there is primarily indirect evidence for such a relationship from studies using performance-based experiments. Indirect support comes from research on the personality trait alexithymia suggesting that difficulties in describing emotions, which implies low emotional clarity (Gohm and Clore, 2000), is related to deficits in the identification of emotions in facial expressions (Parker et al., 2005; Ihme et al., 2014). Self-reported dispositional emotional clarity was not found to be related to emotion differentiation, i.e., the ability to differentiate affective experience into discrete emotional categories such as anger or shame, as derived from scenario-based and event-sampling-based measures of affect (Boden et al., 2013b). In a large sample of women with eating disorders and healthy women, dispositional emotional clarity did not correlate with the ability to identify facial emotions (Wyssen et al., 2019). There is some evidence that self-reported dispositional emotional clarity is associated with an indirect, reaction-time based measure of emotional clarity, i.e., the latency of state affect ratings (Lischetzke et al., 2005). It can be expected that individuals who are in general clear about their emotions should manifest a higher speed at which they identify the type of emotion they experience in a certain moment compared to those who are in general unclear about their emotions. When individuals can easily access and interpret internal emotional cues, they should be able to evaluate their current emotional states comparatively fast. However, dispositional clarity and response times of mood ratings were found to be significantly correlated only when the direct measure was assessed first but not when the indirect measure was assessed first (Lischetzke et al., 2005, 2011). This finding was interpreted as a category activation effect: the reporting on one’s level of emotional clarity could make one’s standing on this dimension salient, which could promote trait-consistent behavior (Lischetzke et al., 2005). It can be assumed that being clear about one’s emotions also goes along with an enhanced efficacy of perception and evaluation concerning external emotional stimuli.

In this context, it should be noted that the associations between self-report and experimental (or behavioral) measures of psychological constructs are in general rather weak. For example, studies on empathy or cognitive control examining the convergence of self-report data and behavioral performance data observed, if any, only small to moderate positive correlations across different measurement types (Melchers et al., 2015; Snyder et al., 2021). One explanation could be that questionnaires measure self-perception, while performance-based measures such as recognition tasks assess abilities. It can be argued also that questionnaires may ask about behaviors and experiences over an extended period of time, whereas the experimental task assesses performance at only one time point (Snyder et al., 2021). Furthermore, self-report and behavioral measures could differ in the breadth of the measurement constructs. A questionnaire may assess general perceptual abilities concerning a wide range of stimuli, while the behavioral task measures the ability to recognize specific stimuli (Melchers et al., 2015).

Attention to and clarity of emotions are considered to be differentially related to wellbeing, emotion regulation, and psychopathology. On the one hand, attention to emotions was found to be largely independent of level of depressive symptoms, social anxiety (Salovey et al., 2002), and subjective well-being (Lischetzke et al., 2012). On the other hand, attention to emotions showed correlations with negative affect (Swinkels and Giuliano, 1995) and neuroticism, i.e., the general tendency to experience negative affective states (Lischetzke et al., 2001; Salguero et al., 2010). Moreover, significant positive relations of attention to emotions were observed with depressive symptomatology and rumination in normal individuals (Extremera and Fernández-Berrocal, 2006; Salguero et al., 2013). Thus, non-clinical individuals who frequently pay attention to their emotions were found to be more engaged in depressive rumination, i.e., repetitive thinking about symptoms of depression and its implications. Excessive monitoring of one’s emotional state can have adverse effects on mood, amplifying depressive symptoms (Salguero et al., 2013). Similarly, patients who give more attention to their emotions while in a depressive episode were less likely to recover compared to those who dedicated less attention to their emotions (Thompson et al., 2013). In sum, findings from previous research show that attending to one’s emotions can be linked to experiences of negative affect and ruminative thought, a core process in the onset and maintenance of depressive symptoms (Gotlib and Joormann, 2010). Attention to own emotions seems to contain maladaptive elements. Individuals who dedicate a lot of attention to their emotions might initiate more easily maladaptive rumination cycles.

In contrast, clarity of emotions has been found to be related to low negative affect (Gohm and Clore, 2002), low neuroticism (Lischetzke et al., 2001), less depressive symptoms (Salguero et al., 2013), less social anxiety (Salovey et al., 2002), enhanced subjective well-being (Lischetzke et al., 2012), and increased dispositional optimism (Extremera et al., 2007). Deficits in emotional clarity seem to be involved in many forms of psychopathology such as depression, anxiety disorders, or substance abuse (Vine and Aldao, 2014). Being clear about one’s emotions seems to have adaptive, mood-protective effects and to promote mental health. An important mechanism underlying these positive effects of emotional clarity may refer to the more efficient use of emotion regulation. It has been argued that a successful identification of emotions frees up cognitive resources that can be used to select and effectively implement emotion regulation strategies (Lischetzke and Eid, 2017). Moreover, emotional clarity has been found to be associated with an internal, stable, and global attributional style for good but not for bad events (Gohm and Clore, 2002). This means that high clarity of emotions goes along with positive, self-protective interpretations of events which can serve as buffers against negative affect and depression (Flynn and Rudolph, 2014).

Negative biases in attention and interpretation are central to cognitive theories of vulnerability to depression (LeMoult and Gotlib, 2019). In the area of bias research in depression, special attention has been given to the perception of emotional expressions in faces because accurate emotion recognition in others is essential to social development and functioning (Bistricky et al., 2011). Dysphoric mood as well as clinical depression have been found to be associated with a negative interpretation bias in the perception of neutral or emotionally ambiguous facial expressions (Leppänen et al., 2004; Beevers et al., 2009). Bouhuys et al. (1995) administered schematic drawn faces to examine the effects of induced negative mood on evaluations of facial expressions in healthy individuals. Even mild degrees of negative mood were associated with the perception of more rejection and sadness in ambiguous faces. Schematic facial expressions, as opposed to photographs of actors, avoid factors that can influence a person’s judgment of emotions, such as the attractiveness, age, and gender of the actor (e.g., Hale, 1998). Judgements of negative emotions in ambiguous schematic facial expressions were found to be better predictors of depressive mood and episodes than judgements of clear (or unambiguous) expressions (Hale, 1998; Bouhuys et al., 1999a). Thus, the PFE task using schematic faces has proved to be useful for the measurement of negatively biased interpretations in non-clinical and clinical individuals (see also Bouhuys et al., 1996, 1999b; Raes et al., 2006). An important factor contributing to negative interpretative bias in the perception of facial displays of emotion is rumination: habitual ruminating about and focusing on depressive symptoms and mood in healthy individuals goes along with a negative bias in the perception of others’ facial expressions (Suslow et al., 2019). In contrast, optimism, a positive attitude characterized by hope and confidence in success, decreases negative interpretations of ambiguous information (Gordon et al., 2016).

Lischetzke et al. (2001) developed a scale for assessing attention to and clarity of emotions, the WEFG (Skalen zur *Wahrnehmung eigener und fremder Gefühle*), with a narrow definition of the constructs which they called *Attention to feelings* and *Clarity of feelings*. Attention to feelings relates here to the habitual tendency to attend to one’s feelings, whereas clarity of feelings refers to the extent to which one’s feelings are in general identified and can be described. Lischetzke et al.’s construct of attention to feelings is more specific compared to that of the Trait Meta-Mood Scale (Salovey et al., 1995) which encloses also valuing and accepting feelings and letting oneself experience them fully. In the scales of Lischetzke et al. (2001) the valence of feelings is not specified, global ratings concerning feeling perception are given.

The present study had two major goals. First, we examined the relation of attention to and clarity of feelings with the efficiency of facial affect perception [research question 1 (RQ1)]. Here we focused on the question whether attention to and clarity of feelings are associated with the speed of evaluative decisions about others’ facial expressions. It was hypothesized that attention to feelings and clarity of feelings are both negatively related to evaluative decision latencies.

Second, we investigated whether attention to and clarity of feelings are differentially linked to negative interpretations of facial expressions (RQ2). Differential links or associations exist if there are differences in the sign or strength of correlations of two (or more) psychological constructs with other variables. We expected that attention to feelings is associated with an increased and clarity of feelings is related to a reduced attribution of negative affect in neutral and ambiguous expressions. As noted above, neutral, and ambiguous faces appear to be the most appropriate facial expressions to detect subtle negative evaluative biases.

A computer-based version of the PFE questionnaire (Bouhuys et al., 1995) was administered to assess perceived emotions in faces. In our analyses, we controlled the effects of other potentially relevant variables such as negative affectivity, i.e., state and trait anxiety, and current level of depressive symptoms, and (verbal) intelligence. Intelligence was considered a control variable since low intelligence has been found to be a risk factor for the development of depression (Lager et al., 2017). Better response to stressors among people with higher intelligence might be one explanation of this finding. Gender was also controlled in our analyses as there is evidence for a female advantage in recognizing facially expressed emotions even though the effect size might be rather small (Connolly et al., 2019; Olderbak et al., 2019).

## Materials and Methods

### Participants

The participants of our study were recruited using online platforms at the University of Leipzig and public notices that were posted in canteens, and student halls of residence but also public libraries and supermarkets. The final sample consisted of 118 healthy individuals (62 women). Ninety-seven of the participants were university students, two were apprentices, 16 were employees, and three were at the time of the study unemployed. All participants were native speakers of German. General exclusion criteria were actual or past presence of psychiatric or neurological diseases, and psychotropic medication use. Prior to the inclusion in the study participants’ visual acuity was checked. All participants read, without any errors, print at least as small as in line 5 when standing 4 ft. from a miniature Snellen eye chart. The mean age of participants was 24.2 years (SD = 4.1; range: 18–35). Their school education had a mean duration of 12.1 years (SD = 0.8). Our study was approved by the ethics committee at the University of Leipzig, Medical Faculty. Informed, written consent to the study was obtained from all participants. All participants received a financial compensation.

### Questionnaires and Tests

The WEFG (Skalen zur *Wahrnehmung eigener und fremder Gefühle*) was administered to assess dispositional attention to and the clarity of one’s own feelings (Lischetzke et al., 2001). Six items assess attention to feelings (“I think about my feelings,” “I pay attention to my feelings,” “I am preoccupied with my feelings,” “I think about how I feel,” “I notice my feelings” and “I focus on how I feel.”) and six items measure clarity of feelings [“I can name my feelings,” “I am confused about what I feel” (inverse item), “I have a hard time describing my feelings” (inverse item), “I know what I feel,” “I have a hard time naming my feelings” (inverse item) and “I am not sure of what I actually feel” (inverse item)] (the English translations of the items were taken from the appendix of Lischetzke and Eid, 2003). The items are rated on four-point frequency scales [ranging from almost never (1) to almost always (4)]. In previous studies, both scores were found to be highly reliable (Lischetzke and Eid, 2003; Lischetzke et al., 2011). In our study, Cronbach’s alpha was 0.90 for both WEFG scales. Evidence has been presented for the factorial and concurrent validity of the WEFG (Lischetzke et al., 2001).

For measuring state and trait anxiety the German version of the State Trait Anxiety Inventory (STAI; Laux et al., 1981) was used. Depressive symptoms of participants were measured with the Beck Depression Inventory (BDI-II; German version: Hautzinger et al., 2006). Verbal intelligence was assessed by the Mehrfachwahl-Wortschatz-Intelligenztest (MWT-B; Lehrl, 2005).

### Perception of Facial Expressions Task

**Figure 1:**
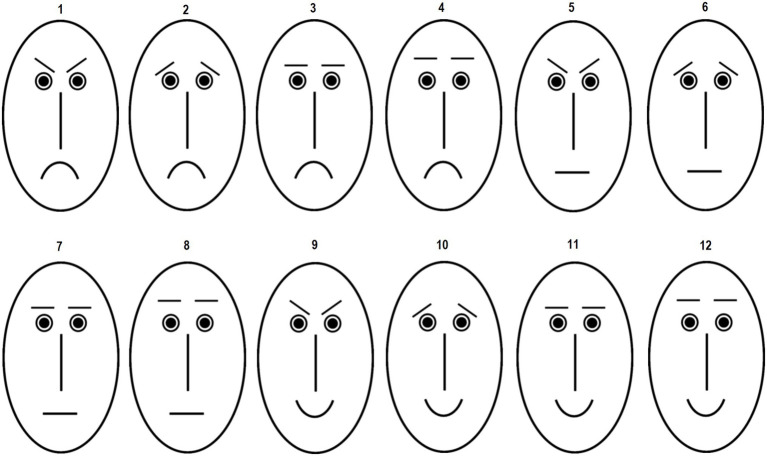
Schematic faces presented in the perception of facial expressions (PFE) task. Faces 1–6 express negative affects. Faces 7 and 8 have a neutral expression and faces 9 and 10 are ambiguous. Faces 11 and 12 express positive affects. Participants had to evaluate each face separately with respect to the degree to which it expresses a certain affect.

The PFE task consisted of 12 schematic oval faces adapted from Bouhuys et al. (1995, 1997). The line drawings were composed from one type of eyes and nose, four eyebrow and three mouth types. Eyebrows consisted of two straight horizontal lines (which differed in their distance to the eyes), and eyebrows with elevated or lowered inner corners. Eyebrows with elevated inner corners have shown to evoke the impression of sadness whereas eyebrows with lowered inner corners are perceived as signaling anger and threat (Hasegawa and Unuma, 2010; Neth and Martinez, 2010). The mouths comprised a straight horizontal line, an upward-curved or a downward-curved mouth line. An upward-curved mouth signals positive mood or happiness whereas a downward-curved mouth is perceived as expressing negative affects such as sadness or disgust (Wierzbicka, 1999; Zhang et al., 2021). The line drawings were black on white background. The schematic faces can be classified as having a negative (faces 1–6), positive (faces 11–12), neutral (faces 7–8), or ambiguous (negative–positive (faces 9–10)) expression (see Figure 1 for facial stimuli).

The stimulus presentation and response registration were realized *via* the program *Inquisit* (Draine, 2004) on a Dell Latitude E6510 with a 15.6-inch screen. Before the start of the experiment, participants were informed on the computer screen that they would see line drawings of faces. Participants were instructed to evaluate the schematic drawings with respect to the degree to which they express the affects sadness, anger, happiness, disgust, surprise, or anxiety. They were asked to make their evaluations according to their personal impression. Participants were told to look at each face carefully and to rate each face on the degree to which it expressed a specific affect using one of five response options: *not at all*, *a little*, *moderately*, *quite a bit* and *very much*. Finally, participants were told that their decision times were being measured.

The faces appeared on the left side of the screen whereas questions (e.g., “Does the face express sadness?”) were presented on the right side of the screen. Images of faces and questions remained on the screen until a response was given. The display size of each face on the screen was 17.3 cm high x 10.5 cm wide. The distance between screen and participants’ eyes was about 60 cm. At the bottom of the screen, the response format was shown. Responses were given on a five-point scale ranging from 1 (*not at all*), 2 (*a little*), 3 (*moderately*), 4 (*quite a bit*) to 5 (*very much*) by pressing the “1,” “2,” “3,” “4,” or “5” key on a keyboard. Participants had to evaluate facial stimuli with respect to six basic affects [*positive*: happiness (Freude); *ambivalent*: surprise (Überraschung), *negative*: anxiety (Angst), anger (Ärger), sadness (Traurigkeit), and disgust (Ekel)]. In total, participants made 72 judgments in the PFE task (12 faces x 6 affect ratings). Facial stimuli were shown in a random order. When a stimulus had been selected six questions were asked consecutively about its expression of affects—in a fixed sequence: (1) sadness, (2) anger, (3) happiness, (4) disgust, (5) surprise, and (6) anxiety. The intertrial interval had a duration of 2000 ms.

### Gender Decision Task

In the gender decision task, we presented faces from the Karolinska Directed Emotional Faces database (KDEF, Lundqvist et al., 1998). The KDEF consists of photographs of young amateur actors (with an age between 20 and 30 years). Participants were shown 35 female and 35 male faces with a neutral expression. The display size of each facial expression was 20 cm high × 14.5 cm wide. All faces were color photographs that were displayed on a black screen. The distance between screen and participants’ eyes was about 60 cm.

The stimulus presentation and response registration were realized *via* Inquisit (Draine, 2004) on a Dell Latitude E6510. The task consisted of six practice trials and 70 experimental trials presented in a fixed random order. The internet-based program *Random*^1^ was used to determine the random sequence of trials. Each trial had the following routine: after the presentation of a fixation cross (shown for 800 ms) a facial stimulus was presented for 1,200 ms. Participants were instructed to view a series of faces and to categorize them by gender as quickly but also as accurately as possible (dichotomous decision: woman or man). They provided their decision by pressing one of two response buttons on the keyboard (“v” for woman or “n” for man). Trials were terminated by the participant’s response. An intertrial interval of 2000 ms separated subsequent trials.

### General Procedure

Testing sessions were conducted individually in a quiet room at the Department of Psychosomatic Medicine and Psychotherapy at the University of Leipzig. The tests were administered in a fixed order. Participants completed the STAI-State, a gender decision task, the WEFG and the STAI-Trait and proceeded with the PFE task. Thereafter, participants were given the MWT-B and the BDI-II.

### Statistical Analysis

The statistical analyses of the PFE data were divided into two parts. First, an overall evaluation score and an overall evaluative decision latency score were computed for each participant (across all affect categories and all facial stimuli). Second, two negative bias scores were calculated from the evaluative data for each participant: (1) judgments of the four negative categories (i.e., sadness, anger, disgust, and anxiety) were averaged over the two neutral facial stimuli (faces 7 and 8): “negative affects in neutral faces,” (2) judgments of the four negative categories (i.e., sadness, anger, disgust, and anxiety) were averaged over the two ambiguous facial stimuli (faces 9 and 10): “negative affects in ambiguous faces.” In addition, we calculated an evaluation score based on negative faces for each participant: judgments of the four negative categories (i.e., sadness, anger, disgust, and anxiety) were averaged over the six negative facial stimuli (faces 1–6): “negative affects in negative faces.” The latter score can help to assess the specificity of associations of attention to and clarity of feelings with perception of negative affect in neutral and ambiguous faces.

Product–moment correlation analysis was used to examine the relationships between WEFG scales, measures of affectivity, intelligence, PFE, and gender decision. To detect a medium effect of *r* = 0.30 with an alpha value of 0.05, one-tailed, and a statistical power of 0.95, the required sample size is 111 (as calculated with the program G*Power 3.1.9.2.; Faul et al., 2009). To investigate gender differences in attention to feelings, clarity of feelings, affectivity, intelligence, and performance in the PFE (evaluative decision latencies and negative bias scores) and the gender decision task (gender decision latencies) *t*-tests for independent samples were applied. As attention to and clarity of feelings were found to correlate with each other in our sample, additional partial correlation analyses were conducted in order to investigate their specific relationships to performance indices in the PFE task. In general, results were considered significant at *p* < 0.05, two-tailed. All calculations were made with SPSS 27.0 (IBM Corp., Armonk, NY, United States), except the comparisons of correlation coefficients (from dependent samples) based on Steiger’s *Z* which were calculated according to Eid et al. (2011) using the free software Psychometrica (Lenhard and Lenhard, 2014).^2^ The majority of correlation analyses were conducted to control confounders and to identify factors associated with the emotional awareness variables or the performance in the PFE task. Six correlation analyses were performed to test our directed hypotheses concerning the relationships of attention to and clarity of feelings with evaluative decision latency (RQ1) and the bias scores “negative affects in neutral faces” and “negative affects in ambiguous faces” (RQ2). To correct for multiple testing, we used the Benjamini–Hochberg procedure (Benjamini and Hochberg, 1995) which leads to a lower rate of false negative results compared to the conservative Bonferroni correction while still controlling the false discovery rate. In this procedure, the observed values of *p* are arranged in ascending order, assigning a rank to each one. To calculate the critical value for each value of *p*, the formula (i/m) * Q is used where *i* refers to the rank of the value of *p*, *m* refers to the total number of tests, and *Q* reflects the chosen false discovery rate. The original values of *p* are compared with the critical values and the largest value of *p* has to be identified that is smaller than the critical value. The tests with value of *p* smaller than the critical value are considered significant according to the Benjamini–Hochberg procedure. Since all hypotheses were directed one-tailed testing was considered appropriate in this case.

Participants in the PFE task are under no particular time pressure to make evaluative decisions (see our instructions—cf. Bouhuys et al., 1997; Hale et al., 1998). Against this background it seems appropriate to exclude only very slow responses or responders from data analysis. Participants gave 8,856 responses in the PFE task. Evaluative decision latencies were in no case below 250 ms. Twenty evaluative decision latencies were above 20 s and were excluded from further data processing. To identify very slow responders, we analyzed overall evaluative decision latencies of all participants. The interquartile range was used to find outliers. Outliers were defined as mean latencies that fell 1.5 interquartile ranges above the third quartile (cut-off score: 5451 ms) or below the first quartile (cut-off score: 939 ms; see Tukey, 1977). Five participants had higher mean overall evaluative decision latencies and were excluded from data analysis so that the final sample consisted of 118 individuals.

In the gender decision task participants gave 8,610 responses. They made 144 classification errors (1.67%). No decision latency was below 250 ms. Gender decision latencies that were considered outlier values, i.e., those values that were 1.5 interquartile ranges above the third quartile or below the first quartile with respect to the individual distribution were discarded (4.8% of the correct answers). Finally, mean gender decision latencies were calculated for correct gender classification responses for each participant.

## Results

### Relationships of Attention to and Clarity of Feelings With Negative Affectivity and Intelligence

As could be expected, attention to feelings was significantly (but moderately) related to clarity of feelings (*r* = 0.19; *p* < 0.05). No correlations were observed between attention to feelings and state anxiety, trait anxiety, depressive symptoms, and intelligence. Clarity of feelings showed significant negative correlations of large effect size with trait anxiety (*r* = −0.52; *p* < 0.001) and depressive symptoms (*r* = −0.50; *p* < 0.001) and a (rather small) negative correlation with state anxiety (*r* = −0.19; *p* < 0.05) while no correlation was found with intelligence (see Table 1).

**Table 1:** Descriptive statistics and product–moment correlations between psychological measures (*N* = 118).

	1	2	3	4	5	6
1. AF	−					
2. CF	0.19^*^	−				
3. STAI-State	0.05	−0.19^*^	−			
4. STAI-Trait	−0.02	−0.52^**^	0.57^**^	−		
5. BDI-II	−0.12	−0.50^**^	0.37^**^	0.61^**^	−	
6. MWT-B IQ	0.00	0.05	0.00	0.00	0.02	−
Mean	2.96	3.17	35.44	39.65	8.39	109.81
SD	0.65	0.61	6.07	9.58	6.31	11.00

*AF, attention to feelings (mean item score); CF, clarity of feelings (mean item score); STAI, State Trait Anxiety Inventory; BDI-II, Beck Depression Inventory; and MWT-B IQ, multiple-choice vocabulary test version B intelligence quotient*.

* *p* *≤ 0.05;*

** *p ≤ 0.001 (two-tailed)*.

According to *t*-tests, women had higher attention to feelings scores (3.09, SD = 0.61) compared to men (2.83, SD = 0.67), *t* (116) = 2.21, *p* < 0.05. No gender differences emerged for clarity of feelings, negative affect measures, and intelligence (all *p*s > 0.10).

### Relationships of Attention to and Clarity of Feelings With Decision Latencies in the Perception of Facial Expressions Task and the Gender Decision Task (RQ1)

In our sample, the decision latencies for the six affect categories were as follows: 4,421 ms (SD = 1,244) for sadness, 3,264 ms (SD = 968) for anger, 2,638 ms (SD = 776) for happiness, 2,641 ms (SD = 741) for disgust, 2,887 ms (SD = 1,020) for surprise, and 3,128 ms (SD = 1,035) for anxiety. Response latencies for the decision how much faces expressed sadness were by far higher than the decision times for the other affects. The mean overall evaluative decision latency (across all affect categories and faces) was 3,163 ms (SD = 801) in the PFE task. The mean overall gender decision latency for correct classification responses was 612 ms (SD = 142) in the gender decision task.

**Figure 2:**
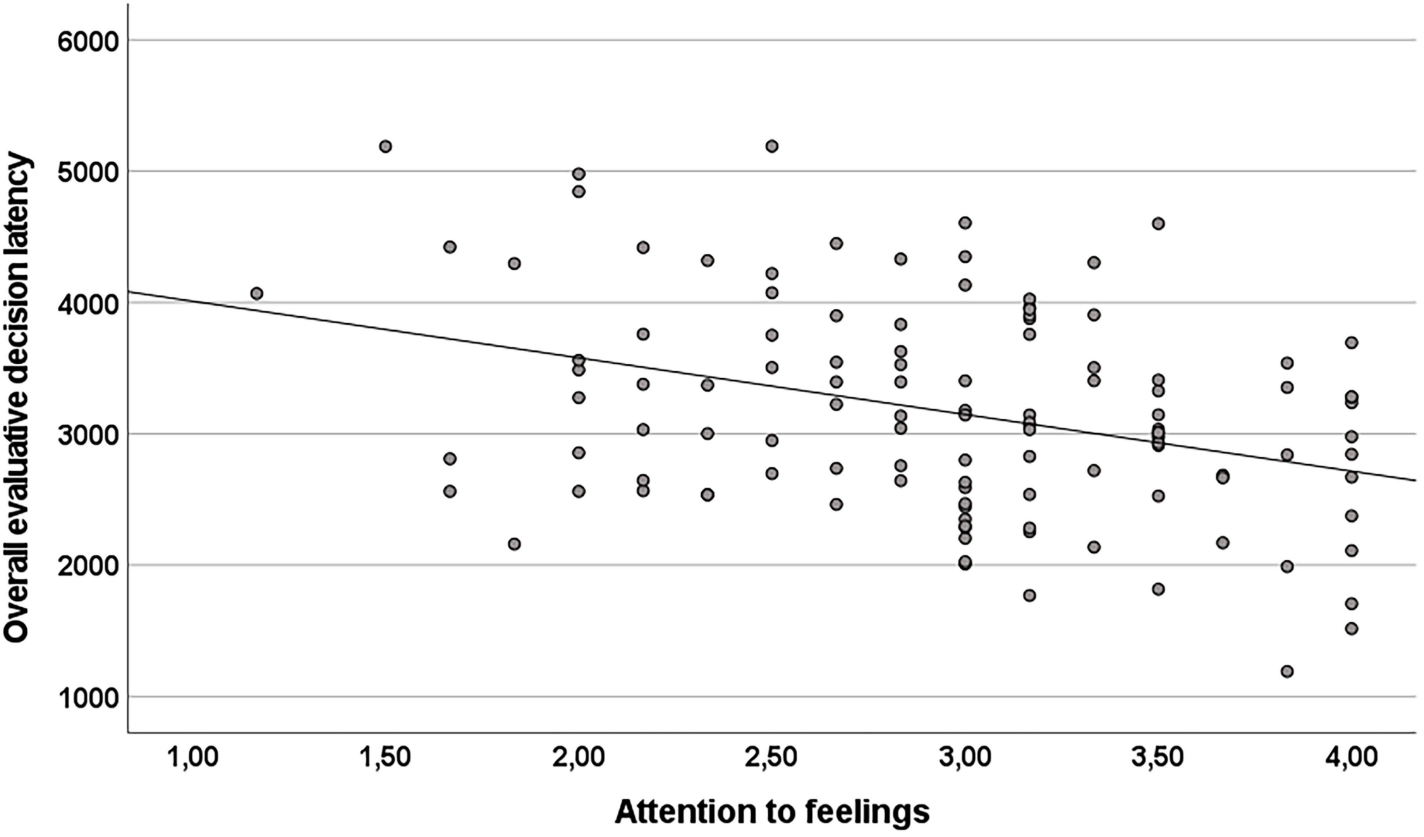
The scatterplot depicts the negative correlation between dispositional attention to feelings as assessed by the WEFG (mean item score) and overall evaluative decision latency (in milliseconds) in the PFE task (*r* = −0.35, *p* < 0.001; *N* = 118).

According to our data, participants’ attention to feelings was negatively correlated with the overall evaluative decision latency in the PFE task (*r* = −0.35; *p* < 0.001, see Table 2; Figure 2). In contrast, there was no significant correlation of clarity of feelings with overall evaluative decision latency (*r* = −0.10; *p* = 0.28). According to Steiger’s *Z* the correlation between attention to feelings and overall evaluative decision latency was significantly stronger than the correlation between clarity of feelings and overall evaluative decision latency in the PFE task (*Z* = −2.2, *p* = 0.01). The correlation between attention to feelings and overall evaluative decision latency remained significant when controlling for clarity of feelings (partial *r* = −0.34; *p* < 0.001). The three measures of negative affectivity (STAI-State, STAI-Trait and BDI-II) and verbal intelligence (as assessed by the MWT-B) were not correlated with overall evaluative decision latency in the PFE task (see Table 2). According to *t*-tests, no gender differences emerged for overall decision latencies in the PFE and the gender decision task (all *p*s > 0.10).

**Table 2:** Correlations of psychometric measures with performance in the PFE task (overall evaluative decision latency, overall evaluation score, and bias scores) and the gender decision task (overall gender decision latency; *N* = 118).

	AF	CF	STAI-State	STAI-Trait	BDI-II	MWT-B IQ
PFE task—overall evaluative decision latency	−0.35^***^	−0.10	0.01	0.09	0.11	−0.10
Overall gender decision latency	0.06	−0.04	0.01	0.08	0.04	−0.08
PFE task—overall evaluation score	0.05	−0.02	0.29^**^	0.10	0.06	−0.25^**^
Negative affect in negative faces	0.02	0.00	0.26^**^	0.08	0.02	−0.24^**^
Negative affect in neutral faces	0.07	−0.08	0.14	0.10	0.04	−0.22^*^
Negative affect in ambiguous faces	0.21^*^	−0.05	0.20^*^	0.06	0.01	−0.24^**^

*AF, attention to feelings; CF, clarity of feelings; STAI, State Trait Anxiety Inventory; BDI-II, Beck Depression Inventory; and MWT-B IQ, multiple-choice vocabulary test version B intelligence quotient*.

* *p* *≤ 0.05;*

** *p* *≤ 0.01;*

*** *p ≤ 0.001 (two-tailed)*.

There were no significant correlations of attention to and clarity of feelings with decision latency in the gender decision task (*r* = 0.06 and *r* = −0.04, see Table 2 for details). The three measures of negative affectivity (STAI-State, STAI-Trait and BDI-II) and verbal intelligence were not correlated with gender decision latency.

### Relationships of Attention to and Clarity of Feelings With Evaluation and Bias Scores in the Perception of Facial Expressions Task (RQ2)

The mean evaluation scores for the six affect categories were: 3.75 (SD = 0.39) for sadness, 3.24 (SD = 0.37) for anger, 2.78 (SD = 0.22) for happiness, 2.44 (SD = 0.44) for disgust, 2.70 (SD = 0.50) for surprise, and 2.89 (SD = 0.58) for anxiety. Thus, sadness and anger were the affects most attributed to the facial expressions. The mean overall evaluation score (across all affect categories) was 2.97 (SD = 0.29).

The correlation analysis revealed that attention to feelings as well as clarity of feelings were not related to the overall evaluation score (see Table 2). In contrast, state anxiety was positively associated with the overall evaluation score. Trait anxiety and level of depressive symptoms did not correlate with the overall evaluation score in the PFE task. Interestingly, intelligence was negatively correlated with the overall evaluation score (see Table 2).

The mean bias score “negative affects in neutral faces” was 3.21 (SD = 0.61), the bias score “negative affects in ambiguous faces” was 2.64 (SD = 0.47), and the evaluation score “negative affects in negative faces” was 3.48 (SD = 0.42).

According to our correlation results, attention to feelings was positively associated with the bias score “negative affects in ambiguous faces” (*r* = 0.21; *p* < 0.05) but not with the other bias or evaluation scores (see Table 2). Clarity of feelings did not correlate with any of the bias or evaluation scores. In contrast, state anxiety showed significant positive correlations with the evaluation score “negative affects in negative faces” and the bias score “negative affects in ambiguous faces” (see Table 2 for details). Trait anxiety and depressive symptoms were not related to the negative bias or evaluation scores. Finally, intelligence was negatively correlated with the two bias scores and the evaluation score (see Table 2). The correlation between attention to feelings and the bias score “negative affects in ambiguous faces” remained significant when controlling for clarity of feelings (partial *r* = 0.23; *p* < 0.05) and when controlling for clarity of feelings, state anxiety and intelligence (partial *r* = 0.22; *p* < 0.05).

According to *t*-tests, no gender differences were observed for the overall evaluation score and the negative bias scores (i.e., “negative affects in neutral faces” and “negative affects in ambiguous faces”) as well as the evaluation score “negative affects in negative faces” in the PFE task (all *p*s > 0.10).

In our study, six correlation tests could be considered as central to answer our two research questions (i.e., the correlations of attention to and clarity of feelings with overall evaluative decision latency, and the bias scores “negative affects in neutral faces” and “negative affects in ambiguous faces”). Thus, the total number of tests was six in the Benjamini–Hochberg correction procedure. When choosing a false discovery rate of 0.05 the largest observed value of *p* (0.0105) smaller than the critical value (0.0166) referred to the correlation between attention to feelings and the bias score “negative affects in ambiguous faces.” Against this background, it can be concluded that two one-tailed tests were significant after α-adjustment: the correlations of attention to feelings with overall evaluative decision latency (*r* = −0.35, observed *p* = 0.0001, critical value: 0.0083) and with the bias score “negative affects in ambiguous faces” (*r* = 0.21, *p* = 0.0105, critical value: 0.0166).

The bias score “negative affects in ambiguous faces” was not related to the overall evaluative decision latency in the PFE task (*r* = −0.01).

## Discussion

In our study, we examined two main research questions concerning central dimensions of individual differences in emotion awareness and perception, dispositional attention to and clarity of one’s emotions. First, we investigated the relation of these dimensions with the efficiency of facial affect perception (RQ1). More specifically, it was tested whether attention to and clarity of one’s own feelings are associated with the speed of affective decisions about others’ facial expressions. Previous research based on self-report measures indicated associations between recognition of and attention to one’s own emotions and recognition of and attention to other people’s emotions (Lischetzke et al., 2001, 2012). Second, the relationships of attention to and clarity of feelings with negative interpretations of neutral and ambiguous emotional facial expressions were examined (RQ2). According to findings from prior studies, dispositional attention to emotions and dispositional emotional clarity are differentially linked to negative affect and wellbeing (e.g., Salovey et al., 2002; Salguero et al., 2013). To answer the research questions, we administered a computer-based version of the PFE test (Bouhuys et al., 1995, 1997) to a sample of healthy individuals. Participants were instructed to rate how much each face expressed a specific emotion according to their personal impression. We employed the WEFG scale of Lischetzke et al. (2001) which measures attention to emotions and emotional clarity using narrow definitions of the constructs. This could have increased the probability to reveal associations between the self-report emotional awareness data and the behavioral performance data in the emotion perception test.

The results of our study corroborate in part our hypothesis, according to which attention to feelings and clarity of feelings are negatively related to evaluative decision latencies (RQ1). The present data indicate that dispositional attention to feelings was negatively associated with the overall evaluative decision latency, whereas dispositional clarity of feelings was unrelated to speed of evaluative ratings. The correlation between attention to feelings and overall evaluative decision time in the PFE was independent from emotional clarity and was significantly higher than the correlation between emotional clarity and overall evaluative decision time. In our study, overall decision latencies did not differ between men and women, and they were not related to state anxiety, trait anxiety, depressive symptoms, or intelligence. Thus, there is evidence that dispositional attention to one’s own feelings is linked to higher speed in deciding how much faces express emotions. Instead, attention to feelings was not related to the speed in categorizing faces by gender. Hence attention to feelings does not go along with a general acceleration of response behavior. It appears that frequently paying attention to one’s own emotions in everyday life may facilitate processing of external emotional information and accelerate evaluative decisions about external stimuli. Findings from psychological, eye-tracking, and neurophysiological research suggest that dispositional attention to one’s feelings goes along with a more rapid allocation of attention resources to and increased neural processing of external emotional information during early stages of perception (Coffey et al., 2003; Fisher et al., 2010; Bujanow et al., 2020). Frequent processing of a specific type of information is known to increase speed of processing (Higgins, 1996). Habitual reflection upon one’s own emotions may enhance efficiency of emotion processing not only in oneself but also of emotional information in others or in the environment. This association between self and other perception may arise from the fact that both processes rely on emotion knowledge (Israelashvili et al., 2019). Emotion concepts represented in language and their accessibility play a constitutive role in emotion perception in general (Lindquist and Gendron, 2013). Individuals who respond quickly may have more accessible emotion concepts. Moreover, according to sensorimotor and body-state simulation accounts of emotion recognition, a key mechanism by which we recognize another individual’s emotion expressed by facial expression could depend on internally simulating the emotional state in ourselves and subsequently identifying or categorizing it (Ross and Atkinson, 2020).

Our data do not confirm the assumption that emotional clarity is negatively related to evaluative decision latency. In the present study, clarity of feelings was unrelated to speed of affective ratings of faces. This was the case even though the self-report measure was given to our study participants before conducting the perception experiment which may have promoted trait-consistent behavior by activation of the salient category. Dispositional clarity upon one’s emotions seems to be independent of the efficiency to make evaluative decisions about external emotional stimuli. Reasons for this could be differences in the assessment objects (self vs. other) and in the constructs’ temporal frames of reference. In the perception task, momentary processes of emotion perception and recognition are measured whereas in questionnaires the assessed abilities to recognize and understand emotions refer to much longer periods of time and require judgments across many situations. Another difference lies in the method of assessment: self-descriptive vs. performance based. Moreover, self-reports of emotional clarity could have been affected by a social desirability bias. Individuals could be confronted with societal demands to act emotionally intelligent which includes competencies in labeling and knowing one’s emotions. Last but not least, dispositional emotional clarity could include personality-related beliefs and positive self-views (Boden et al., 2013b). Some individuals might be convinced to easily gain certainty about their feelings whereas others might remain rather doubtful about their feeling state. The former could behave in a self-protective manner and tend to deny difficulties in the identification and labeling of their emotional experience. This behavior could be relatively independent of their actual recognition abilities.

The second goal of our work was to investigate whether attention to and clarity of feelings are differentially linked to negative interpretations of facial expressions (RQ2). Previous research has shown that attention to emotions is frequently associated with negative affect, depressive symptoms, and rumination (Lischetzke et al., 2001; Extremera and Fernández-Berrocal, 2006; Salguero et al., 2013) whereas emotional clarity has been found to be related to less depressive symptoms, increased well-being, and optimism (Lischetzke et al., 2012; Salguero et al., 2013). Negative biases in interpretation are assumed to be important factors that contribute to cognitive vulnerability to depressive mood and symptoms (LeMoult and Gotlib, 2019). Neutral, and ambiguous facial expressions have been shown to be more sensitive to detect negative evaluative biases than clear or unambiguous expressions (Hale, 1998; Bouhuys et al., 1999a). The findings of our study provide some evidence for a relationship of attention to feelings with negative interpretations of facial expressions and corroborate at least in part our hypotheses. According to our results, attention to feelings was positively linked to the bias score “negative affects in ambiguous faces” but not to the bias score “negative affects in neutral faces” or the evaluation score “negative affects in negative faces.” Contrary to expectation, clarity of feelings was found to be unrelated to all negative bias or evaluation scores. In our investigation, negative bias scores did not differ between men and women, and they were not related to trait anxiety and depressive symptoms. According to our data, state anxiety went along with negatively distorted interpretations of faces for ambiguous expressions. This is in line with prior research showing that state anxiety increases the tendency to perceive negative affect in ambiguous facial expressions (Attwood et al., 2017). In contrast, our results also indicate a negative correlation between intelligence and negative bias scores (for neutral and ambiguous expressions). Thus, we found low intelligence to be related to more perception of negative affect in facial expressions. Interestingly, low intelligence is known to be a risk factor for the development of depression (Lager et al., 2017). The association between dispositional attention to feelings and negative interpretation of ambiguous facial expressions was independent of emotional clarity, state anxiety, and intelligence.

As mentioned above, in the majority of prior studies dispositional attention to emotions was related to negative affect, depressive symptoms and rumination (Lischetzke et al., 2001; Extremera and Fernández-Berrocal, 2006; Salguero et al., 2013). In our study, we found no correlations between attention to emotions and state anxiety, trait anxiety, or level of depressive symptoms but we observed that frequent attending to one’s emotions was specifically linked to the perception of more negative emotions in ambiguous facial expressions. This finding fits with the idea that, in healthy individuals, dispositional attention to feelings could be characterized by subtle negative evaluative tendencies. It has to be noted that in our study there was no association between evaluative decision latencies and perception of negative emotions in ambiguous facial expressions. That means that speed of evaluative decisions and negative evaluative bias (for ambiguous faces) are independently from each other related to dispositional attention to feelings. Faster emotional evaluations and negatively biased evaluations in individuals high in dispositional attention to feelings might rely on different mechanisms or processes. It is interesting to note in this context that in cognitive bias research biases in attention allocation and interpretation have been found to be rather independent predictors of emotional disorders, anxiety symptoms, and depression (Pergamin-Hight et al., 2016; Klein et al., 2018) and, at least in samples of healthy individuals, there is evidence that attentional and interpretation biases can be unrelated (Todd et al., 2016a,b).

How could dispositional attention to feelings be linked to a tendency to interpret ambiguous stimuli negatively? Individuals high in attention to feelings become more frequently aware of their emotions, and they are more often preoccupied with their feelings compared to those low in attention to feelings. Even though their attention could be initially equally directed to positive and negative emotional experiences they might think longer or more intensely about their negative emotions such as anxiety, anger, or sadness than about their positive emotions since the former point to urgent current problems, unachieved goals, or unsatisfied needs. Focusing intensely on or brooding for prolonged periods of time about negative emotions and their causation could make negative information in the environment to some extent more salient than positive information. However, it has to be emphasized that in our study dispositional attention to feelings in healthy individuals was found to be characterized by subtle negative evaluative tendencies which only emerged in stimuli combining conflicting valence information but not in clearly neutral or unambiguously negative stimuli. Attention to feelings in healthy individuals appears not to be associated with a general negative evaluative tendency. Ruminative thought and negative biases in interpretation have long been recognized as components of cognitive vulnerability to depression (Nolen-Hoeksema et al., 2008; Gotlib and Joormann, 2010). Habitual ruminating about depressive symptoms and mood has been found to be associated with a negative bias in the perception of others’ facial expressions (Suslow et al., 2019). Intense monitoring of one’s emotions may adversely affect evaluations of ambivalent social signals and contribute to the development of negative affect.

Some limitations of the present study should be noted. We did not assess participants’ impulsivity in our study. There is evidence that impulsivity shows small to moderate correlations with dispositional attention to feelings (Coccaro et al., 2016). Impulsivity might lead to faster responding. To be able to draw stronger conclusions it is necessary to control impulsivity of participants in future studies on attention to feelings examining reaction times. We included primarily young and well-educated individuals as study participants which clearly limits the generalizability of our findings. The use of schematic emotional faces can be criticized because of their lack of ecological validity (Pinkham et al., 2010). Against this background, it appears appropriate to administer real human facial expressions in future studies on emotion recognition and negative bias in face perception. One promising technique used to examine interpretation biases of real facial expressions relies on morphed faces (e.g., Jusyte and Schönenberg, 2014). Morphing procedures frequently mix two distinct facial expressions (e.g., happy, and angry faces) generating ambiguous facial stimuli containing conflicting emotional information (Maoz et al., 2017). Moreover, we did not assess participants’ ruminative thinking in our study. To further elucidate the relations between attention to emotions, rumination, and perception of emotional expressions, it appears necessary to measure ruminative response styles in future studies.

To conclude, the present results support a relationship between narrowly defined dispositional attention to feelings and efficiency of facial affect perception. It seems that habitually paying attention to one’s own emotions may facilitate processing of external emotional information and accelerate evaluative decisions about external stimuli. In contrast, dispositional emotional clarity, i.e., the extent to which own emotions are identified and understood in everyday life, was found to be unrelated to the speed of evaluative decisions about others’ facial expressions. Preliminary evidence was obtained suggesting that dispositional attention to one’s feelings is associated with negative evaluative tendencies for ambiguous faces. Thus, habitually attending to one’s emotions could be linked to the perception of more negative emotions in ambiguous facial expressions.

## Data Availability Statement

The raw data supporting the conclusions of this article will be made available by the authors, without undue reservation.

## Ethics Statement

The studies involving human participants were reviewed and approved by the Ethics Committee at the University of Leipzig, Medical Faculty. The patients/participants provided their written informed consent to participate in this study.

## Author Contributions

TS and AK conceived and designed the experiment. TS organized the data collection, analyzed the data, and wrote the manuscript with revisions and contributions from AK. All authors contributed to the article and approved the submitted version.

## Funding

We acknowledge support from Leipzig University for Open Access Publishing.

## Conflict of Interest

The authors declare that the research was conducted in the absence of any commercial or financial relationships that could be construed as a potential conflict of interest.

## Publisher’s Note

All claims expressed in this article are solely those of the authors and do not necessarily represent those of their affiliated organizations, or those of the publisher, the editors and the reviewers. Any product that may be evaluated in this article, or claim that may be made by its manufacturer, is not guaranteed or endorsed by the publisher.

## Footnotes

^1^ https://www.random.org/sequences

^2^ https://www.psychometrica.de/korrelation.html
